# Novel pathogenic variants and genes for myopathies identified by whole exome sequencing

**DOI:** 10.1002/mgg3.142

**Published:** 2015-04-08

**Authors:** Jesse M Hunter, Mary Ellen Ahearn, Christopher D Balak, Winnie S Liang, Ahmet Kurdoglu, Jason J Corneveaux, Megan Russell, Matthew J Huentelman, David W Craig, John Carpten, Stephen W Coons, Daphne E DeMello, Judith G Hall, Saunder M Bernes, Lisa Baumbach-Reardon

**Affiliations:** 1 Integrated Cancer Genomics, Translational Genomics Research Institute (TGen)Phoenix, Arizona; 2 Collaborative Sequencing Center, Translational Genomics Research Institute (TGen)Phoenix, Arizona; 3 Center for Bioinformatics, Translational Genomics Research Institute (TGen)Phoenix, Arizona; 4 Neurogenomics, Translational Genomics Research Institute (TGen)Phoenix, Arizona; 5 Section of Neuropathology, Barrow Neurological InstitutePhoenix, Arizona; 6 Division of Neurology, Phoenix Children’s HospitalPhoenix, Arizona; 7 Departments of Medical Genetics and Pediatrics, University of British ColumbiaVancouver, British Columbia, Canada

**Keywords:** Arthrogryposis, *CACNA1S*, central core disease, *COL6A3*, *COL6A6*, *EMD*, exome, muscular dystrophy, myopathy, *RYR1*

## Abstract

Neuromuscular diseases (NMD) account for a significant proportion of infant and childhood mortality and devastating chronic disease. Determining the specific diagnosis of NMD is challenging due to thousands of unique or rare genetic variants that result in overlapping phenotypes. We present four unique childhood myopathy cases characterized by relatively mild muscle weakness, slowly progressing course, mildly elevated creatine phosphokinase (CPK), and contractures. We also present two additional cases characterized by severe prenatal/neonatal myopathy. Prior extensive genetic testing and histology of these cases did not reveal the genetic etiology of disease. Here, we applied whole exome sequencing (WES) and bioinformatics to identify likely causal pathogenic variants in each pedigree. In two cases, we identified novel pathogenic variants in *COL6A3*. In a third case, we identified novel likely pathogenic variants in *COL6A6* and *COL6A3*. We identified a novel splice variant in *EMD* in a fourth case. Finally, we classify two cases as calcium channelopathies with identification of novel pathogenic variants in *RYR1* and *CACNA1S*. These are the first cases of myopathies reported to be caused by variants in *COL6A6* and *CACNA1S*. Our results demonstrate the utility and genetic diagnostic value of WES in the broad class of NMD phenotypes.

## Introduction

Myopathies and muscular dystrophies can be classified into a large heterogeneous subgroup of neuromuscular diseases (NMDs) that are primarily associated with dysfunction of muscle fibers. Identifying the genetic cause of myopathies can be challenging as symptoms overlap and numerous genetic defects in many genes may underlie the clinical pathology of disease. While symptoms can direct successful genetic diagnosis through testing of single genes or small panels of genes, they may also lead to costly, time-consuming, and often unsuccessful attempts at genetic diagnosis. Next-generation sequencing (NGS) can greatly improve the ability to identify pathogenic variants with a single, timely, affordable assay and is beginning to revolutionize genetic testing (Ng et al. 2009, 2010). We thus applied WES to congenital and childhood genetic diagnostic odyssey cases of myopathy/muscular dystrophy (MD). We provide a clinical description of six cases (Table1) and describe the candidate pathogenic variants identified in each (Table2). We first present three cases with Collagen 6 (Col6) myopathies which all carry an identical pathogenic variant in *COL6A3* (OMIM# 120250). Importantly, we provide evidence that variants in *COL6A6* likely result in myopathy. Next, we present a case with a phenotype very similar to two of the Col6 myopathy cases, but instead was found to have Emery–Dreifuss Muscular Dystrophy (EDMD) caused by a novel variant in *EMD* (OMIM# 300384) at a known pathogenic genomic position. Finally, we present two cases with calcium channelopathies. We present a dominant case of central core disease (CCD) caused by an insertion in *RYR1* (OMIM# 180901), followed by evidence for the first *CACNA1S* (OMIM# 114208)-related congenital myopathy with ophthalmoplegia. Our results shed light on the genetic etiology of several related myopathy cases and provide evidence for the effectiveness of WES in aiding in resolving the genetic diagnosis.

**Table 1:** Case phenotype summary

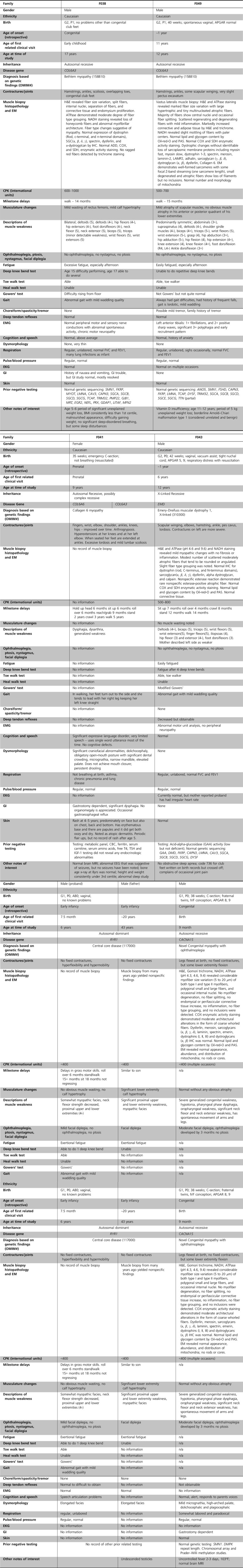

**Table 2:** Novel myopathy pathogenic variants

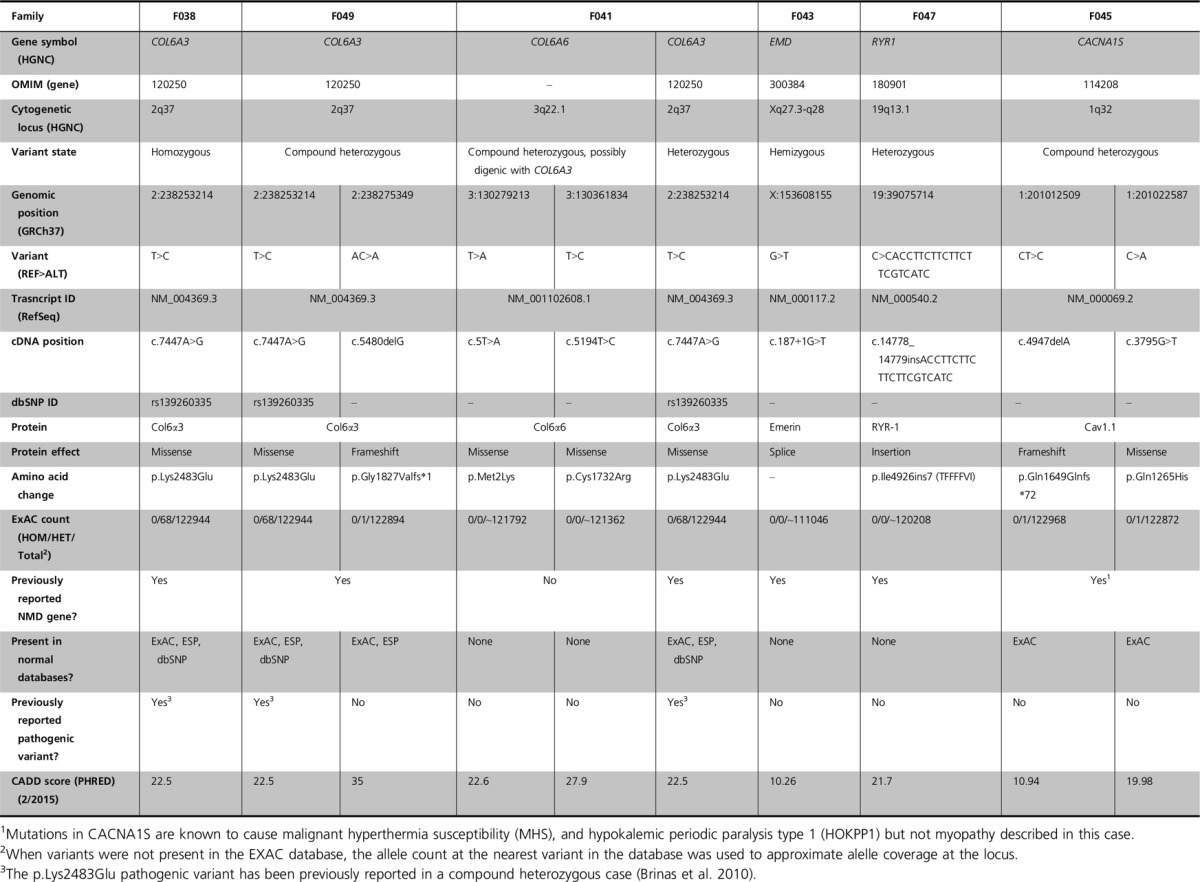

## Materials and Methods

### Patient recruitment and sample collection

Children with clinically diagnosed myopathies and relevant family members were recruited and consented for participation in our research study according to our current Western Institutional Review Board approved protocol (#20120951). Whole blood samples were sent directly to the Dorrance Clinical Laboratory at the Translational Genomics Research Institute (TGen), a CLIA-certified laboratory. DNA was isolated from whole blood and quality and quantity were determined by NanoDrop (ThermoFisher Scientific, Waltham, MA) and Qubit DNA (Life Technologies, Grand Island, NY) assays.

### Whole exome library preparation and sequencing

Whole genome (WG) libraries were prepared according to the manufacturer’s protocols using a TruSeq Library Preparation kit (Illumina, San Diego, CA) or a Library Preparation kit (Kapa Biosystems, Wilmington, MA). DNA quantity and quality were determined by analysis on a High Sensitivity DNA Bioanalyzer chip (Agilent Technologies, Santa Clara, CA). Double-stranded DNA concentrations were obtained using a Qubit Hi-sensitivity DNA assay kit (Life Technologies). WG libraries were pooled and exome enrichment was performed using a modified TruSeq Exome Enrichment kit (Illumina) protocol, where all amplification of exome-enriched libraries was performed using the Kapa Biosystems’ library amplification kit. The enriched libraries were quantitated and qualified using Qubit and Bioanalyzer chip assays as described above. The enriched exome libraries were clustered using the Illumina cBot. Sequencing was performed on Illumina HiSeq 2000/2500 systems using Illumina SBS Kit v3 with 83 × 83 or 100 × 100 base pair (bp) paired-end reads with single index reads of 7 bp according to the manufacturer’s protocols.

### Bioinformatic analysis

Raw sequence data were converted to FASTQ files using Illumina’s BCL Converter tool which were aligned to build 37 of the human reference genome (http://www.ncbi.nlm.nih.gov/projects/genome/assembly/grc/human/) using the Burrows-Wheeler Alignment tool (BWA) (Li and Durbin 2009) and sorted with SAMtools (Li et al. 2009) to create binary sequence (BAM) files. PCR duplicates were flagged for removal using Picard (http://picard.sourceforge.net), which was also used to evaluate other metrics such as coverage and GC metrics. Figure S1 displays basic sequencing coverage metrics.

Variants were called and BAM files were insertion/deletion realigned and recalibrated using The Genome Analysis Toolkit (GATK) (McKenna et al. 2010). The variant call files were annotated with information from the Genetic variant annotation and effect prediction toolbox (SnpEff) (Cingolani et al. 2012), then further annotated with a custom in-house annotation interface tool with information from numerous databases such as ClinVar (www.ncbi.nlm.nih.gov/clinvar/), Polyphen-2 (Adzhubei et al. 2010), FATHMM (Shihab et al. 2013), SIFT (Kumar et al. 2009), Clinical Genomic Database (Solomon et al. 2013), and The National Heart, Lung, and Blood Institute’s GO Exome Sequencing Project (ESP) (ESP 2013). Allele counts from ExAC (Exome_Aggregation_Consortium 2014) and CADD (Kircher et al. 2014) scores were also obtained. Variants were custom sorted for each family based on inheritance patterns and by annotations. Suspect variants classified as “likely pathogenic” or “pathogenic” based on ACMG guidelines (Richards et al. 2008) were clinically confirmed by Sanger sequencing at GeneDX (Cases 2–6) or to ARUP Laboratories (Case 1).

## Results

### Collagen 6 myopathies

#### Case 1. F038 *COL6A3*

**Figure 1:**
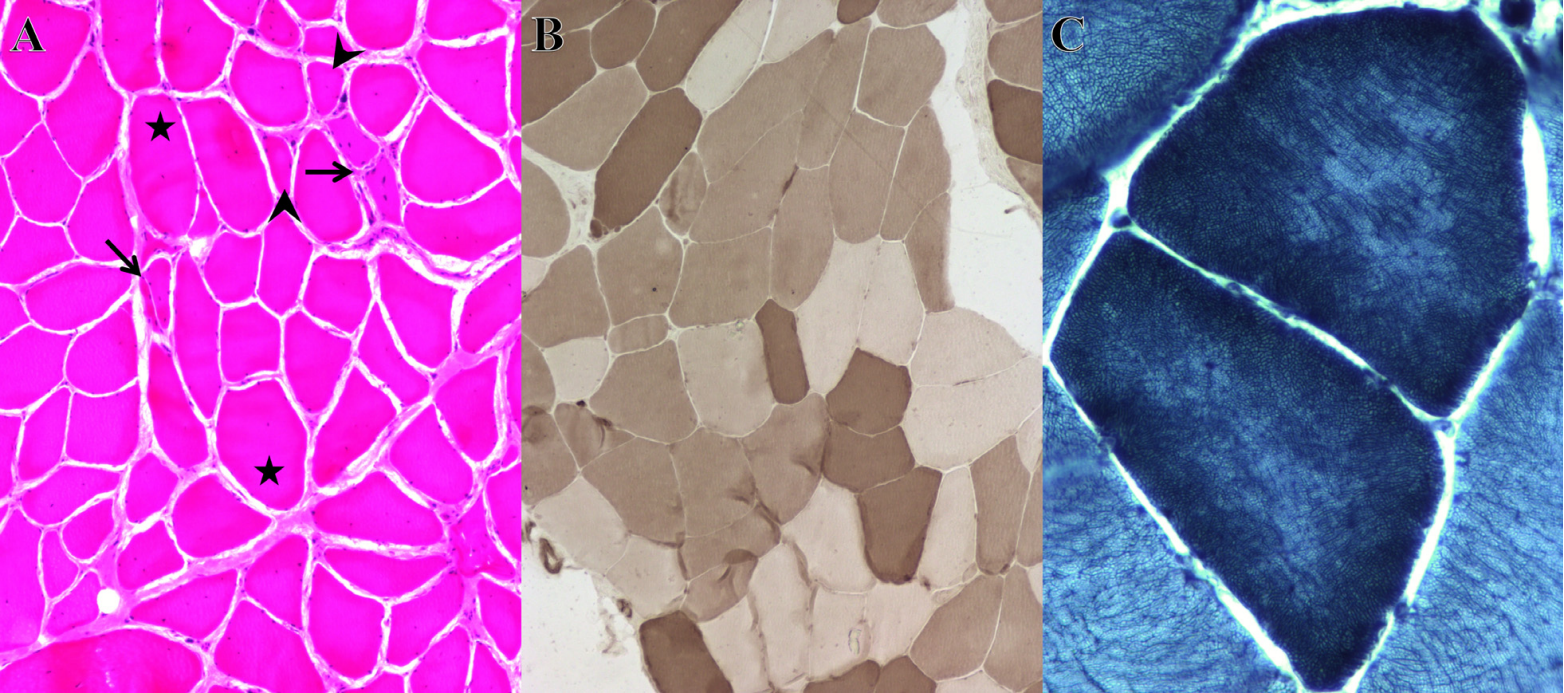
Histopathology images of frozen muscle biopsy cross sections from the affected male child in F038 carrying a homozygous p.Lys2483Glu *COL6A3* pathogenic variant. (A) H&E stain reveals atrophic (arrowheads), hypertrophic (stars), and split fibers (arrows). Also present are internal nuclei and connective tissue proliferation. Magnification = 100×. (B) ATPase reaction stain at pH 4.6 demonstrating that both fiber types are affected by hypertrophy and atrophy as well as mild fiber type grouping. Magnification = 100×. (C) NADH oxidative enzyme reaction stain demonstrating myofibrillar architectural disarray (moth-eaten fibers). Magnification = 400×.

In Family 38, a male child of Canadian descent was born at term with club feet but no additional problems (Table1). He walked somewhat late and since has had a consistently mild abnormal gait. At the age of 5–6 years, he underwent a period of weight loss and lipoatrophy, and at age 17 had a body mass index (BMI) in the first centile, and continued to have a malnourished appearance and difficulty gaining weight. From the age of 8 to age 17, he complained of significant weakness and fatigue. He displayed mild progressive proximal thoracic scoliosis and had significant but nonfixed bilateral lower extremity contractures of hamstrings, ankles, and feet with overlapping toes (Table1). He had no history of skin rash. His respiration and cardiac function were normal. Testing revealed a consistent mild elevation of CPK. Electromyography (EMG) suggested chronic motor neuropathy. Abnormalities revealed by muscle biopsy histology included size variation, split fibers, internal nuclei, connective tissue proliferation, and endomysium proliferation (Fig.1A). ATPase staining revealed moderate Type I and Type II grouping (Fig.1B). NADH staining revealed moth-eaten fibers (Fig.1C). The overall histological diagnosis was abnormal myofibrillar architecture with moderate fiber type grouping of unknown etiology. Genetic sequencing of 15 NMD genes was normal (Table1). Over his 17 years of life, his diagnoses included Charcot–Marie–Tooth disease, myopathy, Pompe disease, MD, limb-girdle muscular dystrophy (LGMD), and spinal muscular atrophy, but without genetic confirmation. There is no other known history of NMD in the family.

**Figure 2:**
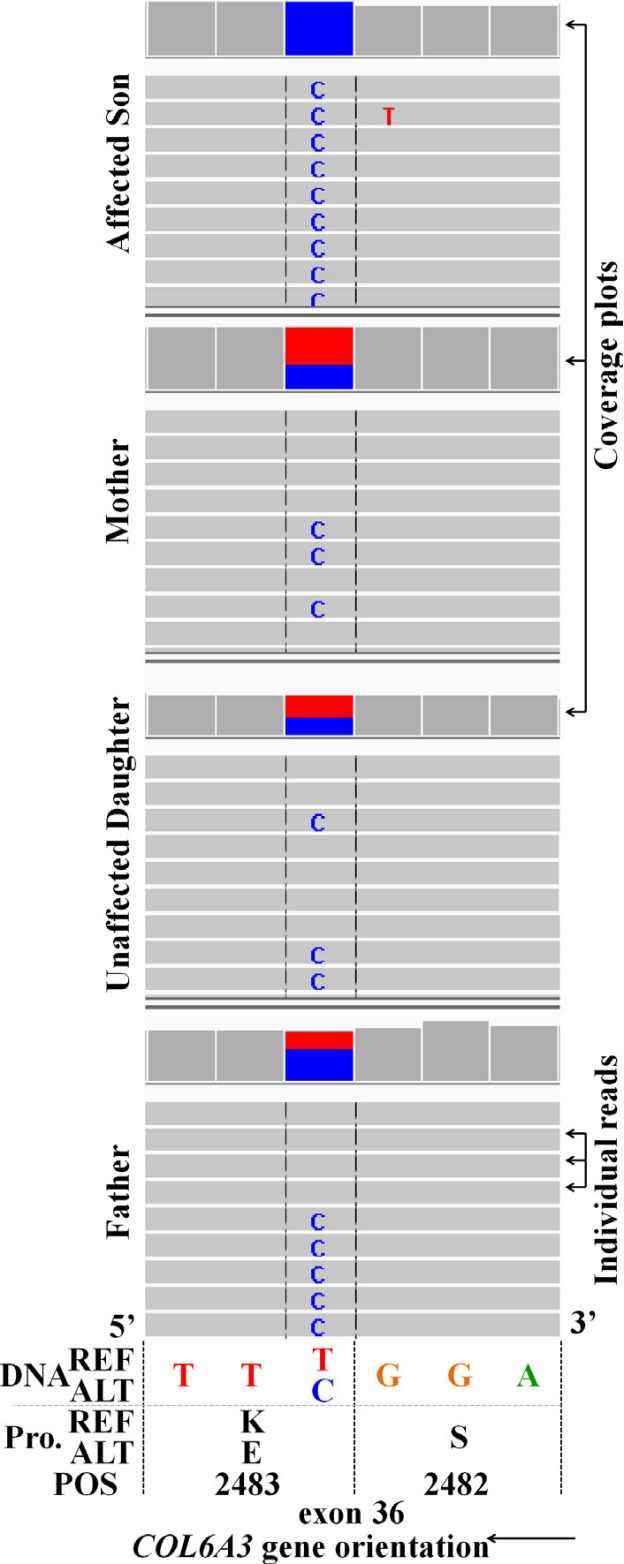
Integrated genomics viewer (IGV) screenshot of F038 WES results of the *COL6A3* c.7447A>G missense p.Lys2483Glu pathogenic variant. The affected son is homozygous for the T>C variant and all other family members represented are heterozygous. Note the DNA reference shows the complementary DNA sequence relative to the gene orientation. DNA reference (REF) based on hGRC37. Col6*α*3 transcript for protein position (Pro. POS) = NM_004369.

**Figure 3:**
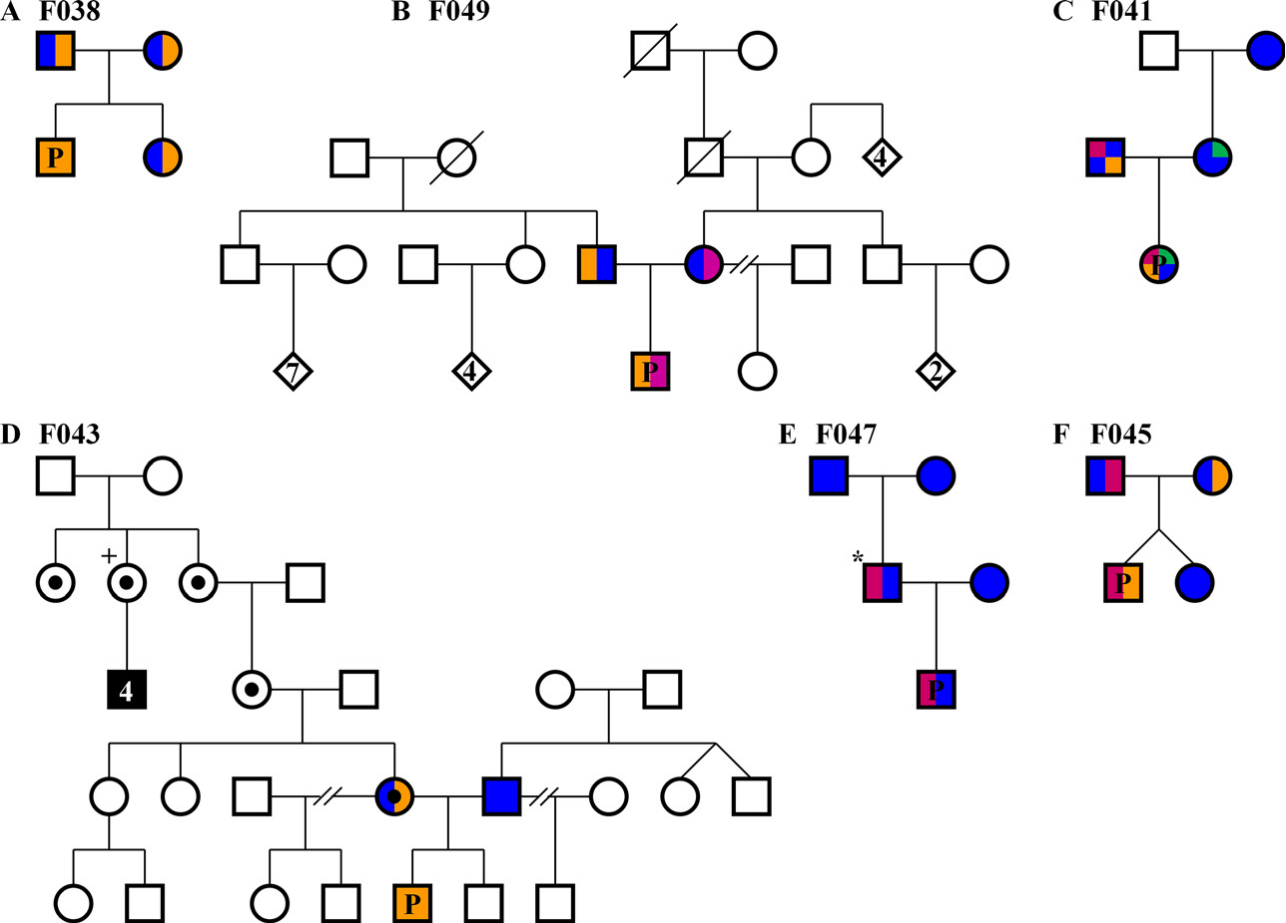
Pedigrees of families. Individuals with color filled symbols underwent exome sequencing. P, proband described in this study. 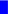 Represents wild-type alleles for the indicated gene in all pedigrees. (A) Family 38 pedigree. 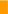 *COL6A3* recessive c.7447A>G variant. (B) Family 49 pedigree. 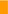 *COL6A3* recessive c.7447A>G variant. 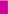 *COL6A3* recessive c.5480delG variant. (C) Family 41 pedigree. 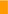 *COL6A3* recessive c.7447A>G variant. 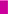 *COL6A6* recessive c.5T>A variant. 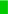 *COL6A6* recessive c.5194T>C variant. (D) Family 43 pedigree. 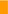 *EMD* recessive c.187+1G>T splice variant. + Required pacemaker and had four affected sons (black fill). ● Known obligate carrier. (E) Family 47 pedigree. 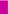 *RYR1* dominant c.14778_14779insACCTTCTTCTTCTTCGTCATC variant. *De novo event. (F) Family 45 pedigree. 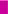 *CACNA1S* recessive c.4947delA variant. 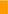 *CACNA1S* recessive c.3795G>T variant.

WES was performed on samples from the parents, proband, and sibling and revealed a rare homozygous missense variant in *COL6A3* (g.chr2:238253214T>C, c.7447A>G, p.Lys2483Glu, NM_004369.3) in the proband (Fig.2 and Table2). The variant was clinically confirmed by Sanger sequencing (data not shown). Each parent and the unaffected female sibling carried this variant in the heterozygous state (Figs.2, 3A). The CADD score for this variant is 22.5. This variant is present in The Single Nucleotide Polymorphism Database (dbSNP) (rs139260335) (Sherry et al. 2001), and ExAC variant databases, but never in the homozygous state. This p.Lys2483Glu variant has been previously identified as pathogenic by Brinas et al. (2010) in a compound heterozygous state, but this is the first homozygous case to our knowledge.

#### Case 2 – F049 *COL6A3*

In Family 49, a male child was born as the product of a 24-year-old, gravida 2 para 1 mother. There were no known complications or health issues at birth. He began walking somewhat late at 14–15 months of age and has always had some gait difficulties (Table1). He was evaluated at the age of 12 years and complains of fatigue and has been unable for several years to keep up with his peers physically. He experienced recent unexplained significant weight loss of 5 kg between the age of 11 and 12. He has no history of rashes or skin abnormalities. He was suspected of having respiratory insufficiency, but had forced expiratory volume 1 (FEV1) and forced volume vital capacity (FVC) of 100 and 105% of predicted. His cognition was normal. In-depth cardiac evaluation was normal. He demonstrated contractures of his hamstrings and ankles and previously wore orthotics to mitigate further ankle contractures. He had no upper extremity contractures but had some protuberance of bilateral scapula. Examination of his strength on multiple occasions by multiple physicians revealed 3–4/5 weakness in all limb and girdle muscles (Table1). He had easily obtainable reflexes, but nerve conduction studies at the age of 9 identified fibrillations and sharp waves in the anterior tibialis. Extensive muscle biopsy histology revealed marked fiber size variation, with large hypertrophic and tiny multinucleated atrophic fibers with a majority showing central nuclei and occasional fiber splitting. Scattered regenerating and degenerating fibers were detected. Both Hematoxylin and Eosin (H&E) and trichrome stains revealed markedly increased connective and adipose tissue (Table1). Of particular interest was the presence of Col6 staining of the endomysial connective tissue and sarcolemmal staining. However, the specific Col6 proteins detected were not reported. NADH reveals slight mottling of fibers with paler centers. Electron microscopy (EM) demonstrated some focal Z-band streaming involving only one sarcomere length. Serial CPK evaluations revealed mildly elevated values in the 500-700 range. His parents and female half sibling are unaffected and no other family history of similar disease was present (Fig.3B).

**Figure 4:**
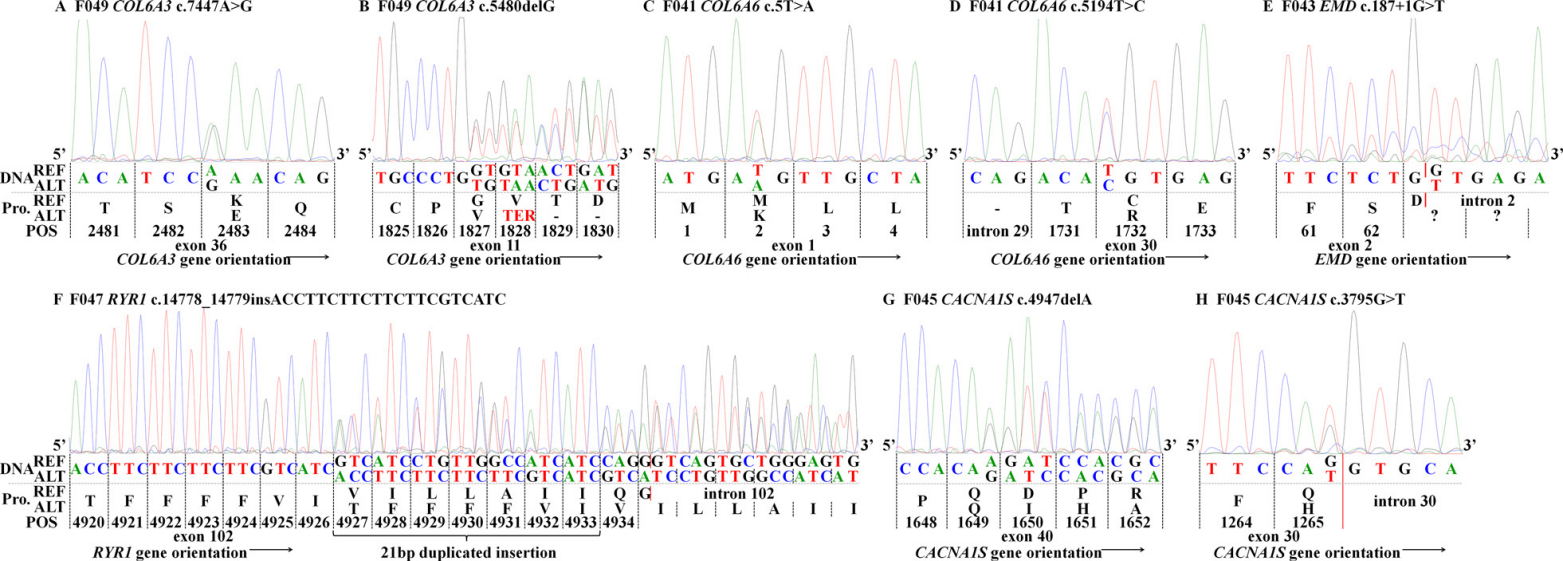
Sanger sequencing traces examples of each pathogenic variant reported from GeneDX clinical confirmation. DNA reference (REF) based on GRCh37. (A) F049 affected male child heterozygous *COL6A3* c.7447A>G recessive missense p.Lys2483Glu pathogenic variant. This trace is also similar to the results found in the F041 affected proband (*data not shown*). (B) F049 affected male child heterozygous *COL6A3* c.5480delG recessive frameshift p.Gly1827Valfs*1 pathogenic variant. (C) F041 affected female child heterozygous *COL6A6* c.5T>A recessive missense p.Met2Lys likely pathogenic variant. (D) F041 affected female child heterozygous *COL6A6* c.5194T>C recessive missense p.Cys1732Arg likely pathogenic variant. (E) F043 affected male child hemizygous *EMD* c.187+1G>T recessive pathogenic variant. (F) F047 affected male child heterozygous *RYR1* c.14778_14779insACCTTCTTCTTCTTCGTCATC dominant duplicated insertion p.Ile4926ins7 (TFFFFVI) pathogenic variant. (G) F045 affected male child heterozygous *CACNA1S* c.4947delA recessive frameshift p.Gln1649Glnfs*72 pathogenic variant. (H) F045 affected male child heterozygous *CACNA1S* c.3795G>T recessive missense p.Gln1265His pathogenic variant. Transcripts used for protein position (Pro. POS) for: Col6*α*3 = NM_004369; Col6*α*6 = NM_001102608; Emerin = NM_000117; RYR-1 = NM_000540; Cav1.1 = NM_000069. Red solid vertical bars represent splice sites. Dashed black vertical bars represent triplet codon reading frame.

WES revealed the same *COL6A3* pathogenic variant (g.chr2:238253214T>C, c.7447A>G, p.Lys2483Glu) identified in the first case, but only in the heterozygous state in case 2 (Figs.3B, 4A, and Table2). A second *COL6A3* frameshift variant (g.chr2:238275349AC>A, c.5480delG, p.Gly1827Valfs*17, NM_004369.3) was also identified in the proband. This variant has been reported in a single allele in ExAC. It is predicted to result in truncation of the protein, and likely does not result in translation of protein due to nonsense-mediated decay (NSMD) (Baker and Parker 2004). Variants were clinically confirmed by Sanger sequencing (Fig.4A and B). The *COL6A3* p.Lys2483Glu pathogenic variant was inherited from the father and the p.Gly1827Valfs*17 pathogenic variant inherited from the mother (Fig.3B). Our case is very similar to the case reported by Brinas et al. (2010) which had the p.Lys2483Glu variant with a frameshift pathogenic variant.

#### Case 3. F041 *COL6A6* and *COL6A3*

In a third family, an infant female child was discovered during an ultrasound to have arthrogryposis. She was born prematurely at 35 weeks by emergency C-section. At birth, her APGAR score was 1. She was not breathing and was resuscitated. She presented further with micrognathia, dysphagia, dysarthria, and generalized hypotonia (Table1). A feeding tube was implanted at 6 weeks of age. She has had significant milestone delays. She began to hold her head up, sit, smile, laugh, and roll over by ∼6 months. She began to reach and grab for things at ∼9 months. She began to stand at 2 years and began to crawl at 3 years of age. At the age of 5 years, she began walking. She has very limited speech. She has consistent muscle weakness of all muscle groups that does not appear to be improving or progressing. At the time of this study, the child was 9 years of age. She has contractures of the fingers, wrist, elbow, shoulders, ankles, knees, and hips. Contractures present from birth have improved somewhat over the years (Table1).

This family, including the proband, her parents, and her maternal grandmother were enrolled in our study. WES revealed the presence of the same *COL6A3* pathogenic variant (g.chr2:238253214T>C, c.7447A>G, p.Lys2483Glu) (Brinas et al. 2010) but only in a heterozygous state in the proband (inherited from her father) with no second pathogenic variant in *COL6A3* detected (Fig.3C). Interestingly, we identified compound heterozygous variants in *COL6A6* in the proband, including a heterozygous missense g.chr3:130279213T>A, c.5T>A, p.Met2Lys (NM_001102608.1) variant inherited from her father and a heterozygous missense g.chr3:130361834 T>C, c.5194T>C, p.Cys1732Arg (NM_001102608.1) variant from her mother (Fig.3C and Table2). All three *COL6* variants in the proband were clinically confirmed by Sanger sequencing (Fig.4C and D). The maternal grandmother did not carry any of these variants (Fig.3C). The *COL6A6* variants have not been identified in dbSNP, ESP or ExAC, and received CADD scores of 22.6 (p.Met2Lys) and 27.9 (p.Cys1732Arg). While CADD scores, which take into account many predictors of pathogenicity, suggest these pathogenic variants are damaging, not all predictors suggest pathogenicity. The Residual Variation Intolerance Score (RVIS) of −0.11 (ALL 0.01% category) for the *COL6A6* gene as a whole compared to all other genes, is only very slightly intolerant of functional variation (Petrovski et al. 2013). Polyphen2 (Adzhubei et al. 2010) ranks both *COL6A6* variants as probably damaging with scores of 0.967 and 1.00 for the p.Met2Lys and p.Cys1732Arg variants, respectively. While we believe that the *COL6A6* variants are likely pathogenic, functional studies are warranted and our interpretation must be considered carefully until further *COL6A6* pathogenic variants are reported.

### Emery–Dreifuss muscular dystrophy

#### Case 4. *EMD* F043

We evaluated a fourth case with similar clinical characteristics to the first two *COL6A3* cases. The male child was born at full term with mild complications with jaundice and wrapping of the umbilical cord around his neck and respiratory distress (Table1). He began walking at 14 months and at ∼4 years old was a toe walker. He was seen at age 6 for abnormal gait, slowly progressing extremity weakness in his feet and ankles, fatigue on excursion, and equinus foot deformity. Further evaluation at 11 years of age noted bilateral winged scapulae, rather significant progressive contractures at his elbows, moderate hamstring contractures, and moderate flexor contractures of his ankles, but no scoliosis (Table1). He had a somewhat unusual gait with a mild waddling quality. He did not demonstrate any neck flexor/extensor or significant proximal weakness in his shoulder girdles. He did have clear distal weakness in foot dorsiflexors and decreased but obtainable deep tendon reflexes. Recent echocardiogram (EKG) and Holter monitoring revealed no structural heart disease, cardio myopathy, or arrhythmia. Muscle biopsy demonstrated mild myopathic changes without inflammation or significant fibrosis (Table1). CPK values were slightly elevated and ranged from 500 to 800. EMG excluded a peripheral neuropathy and was suggestive of myopathy. His working diagnosis was an undetermined form of LGMD. Initially, no family history of related NMD was noted by the family.

The proband and his parents were enrolled in our study and WES revealed a novel pathogenic splice variant in *EMD* at g.chrX:153608155G>T, c.187+1G>T (NM_000117.2) for which the mother is heterozygous and the affected son is hemizygous (Fig.3D and Table2). This variant was clinically confirmed by Sanger sequencing (Fig.4E). This c.187+1G>T splice variant has not been identified in dbSNP, or ExAC, but a c.187+1G>A splice variant has been reported to cause EDMD (Yates et al. 1999). After genetic diagnosis of the proband, several male members of the maternal extended family were identified that had previously been diagnosed and genetically confirmed with EDMD or cardiac conduction defects suggesting this variant is X-linked recessive. However, the sister of the maternal great grandmother required a pacemaker, possibly suggestive of a mild dominant effect or haploinsufficiency in heterozygous females (Fig.3D).

### Calcium channel myopathies

#### Case 5. *RYR1* F047

In F047, a male child was evaluated beginning at 7.5 months of age with delays in gross motor skills. He was not noted of having any problems at birth and went home from the hospital at a few days of life (Table1). He had some milestone delays and was just beginning to roll over at ∼6 month of age and began to walk at 15 months. He has had some speech articulation problems, but cognitive skills are described as normal. When examined at age 5 years of age he had somewhat elongated myopathic facies with mild facial diplegia. He fatigues somewhat more easily than his peers. He had no fixed contractures or obvious muscle wasting. He was able to do a single deep knee bend with difficulty. He rose from the floor with a significant Gowers’ maneuver. His walking gait was abnormal, manifested by a mild waddling quality and his running gait was also abnormal. His neck flexor strength is decreased. Examination of proximal strength in his upper and lower extremities revealed 4-/5 weakness (Table1). His deep tendon reflexes were difficult to obtain. Approximately a year later at age 6 years, his evaluation was much the same, but it was noted that he was able, with difficulty, to walk on his toes, but was unable to walk on his heels. Also noted was a significant degree of hyperflexibility and hypermobility.

The father, age 43 at the time of this study, had a very similar early childhood history. He underwent an extensive neuromuscular evaluation approximately 20 years ago. The father’s evaluation included EMG, normal plasma CPK, and a muscle biopsy which yielded nonspecific results. The father has a history of having generalized fatigue, and exertional weakness. He had no significant history of medical problems other than undescended testicles. At the time of our current study, he was reexamined and had clear evidence for significant proximal weakness in his upper and lower extremities, and could not do a single deep knee bend unaided (Table1). He also had an elongated face with diplegia and myopathic looking facies. He exhibited significant lower extremity calf hypertrophy. The mother had no known neuromuscular disorder.

**Figure 5:**
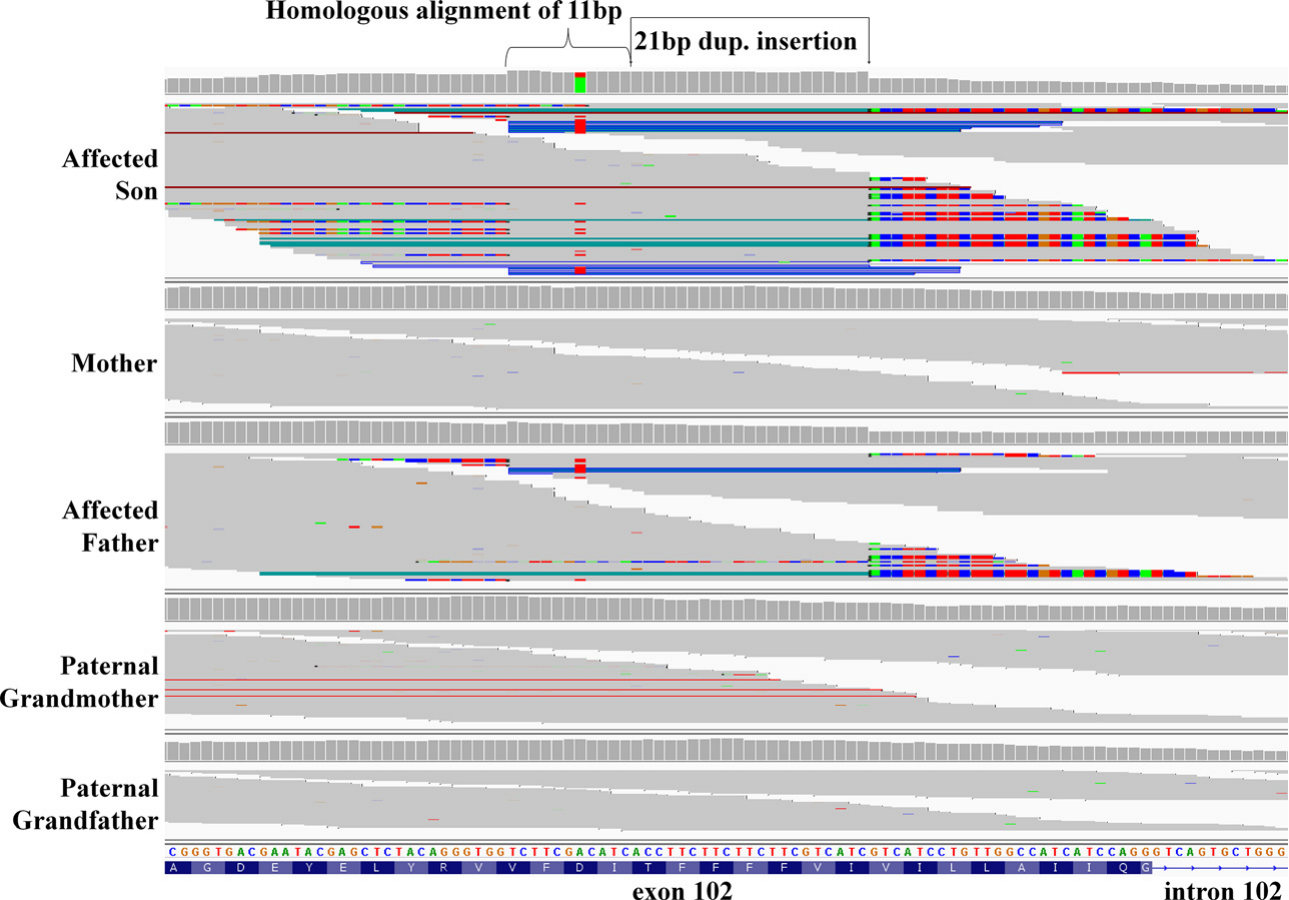
IGV screenshot of the F047 WES results demonstrating a novel 21 bp duplicated *RYR1* c.14778_14779insACCTTCTTCTTCTTCGTCATC dominant in frame p.Ile4926ins7 (TFFFFVI) pathogenic variant. Note the homologous alignment of 11 bp causing the insertion to appear to be 32 bp in length. The insertion is present in the affected son and father but not in the paternal grandparents demonstrating a *de novo* dominant event in the father. RYR-1 transcript = NM_000540.

The boy, his parents, and paternal grandparents were enrolled in our study and WES revealed a novel small in-frame 21 bp duplicated insertion in *RYR1* beginning at g.chr19:39075714, c.14778_14779insACCTTCTTCTTCTTCGTCATC, p.Ile4926ins7 (TFFFFVI) (NM_000540.2) (Fig.5) in the proband and his affected father, but not present in either paternal grandparent indicating that the pathogenic variant is *de novo* in the father (Figs.3E, 5). This variant was clinically confirmed by Sanger sequencing (Fig.4F). This insertion has not been identified in dbSNP, or ExAC (Table2). Pathogenic variants in *RYR1* are the most common cause of CCD (Wu et al. 2006), consistent with the phenotype of the father and affected son.

#### Case 6. F045 *CACNA1S*

**Figure 6:**
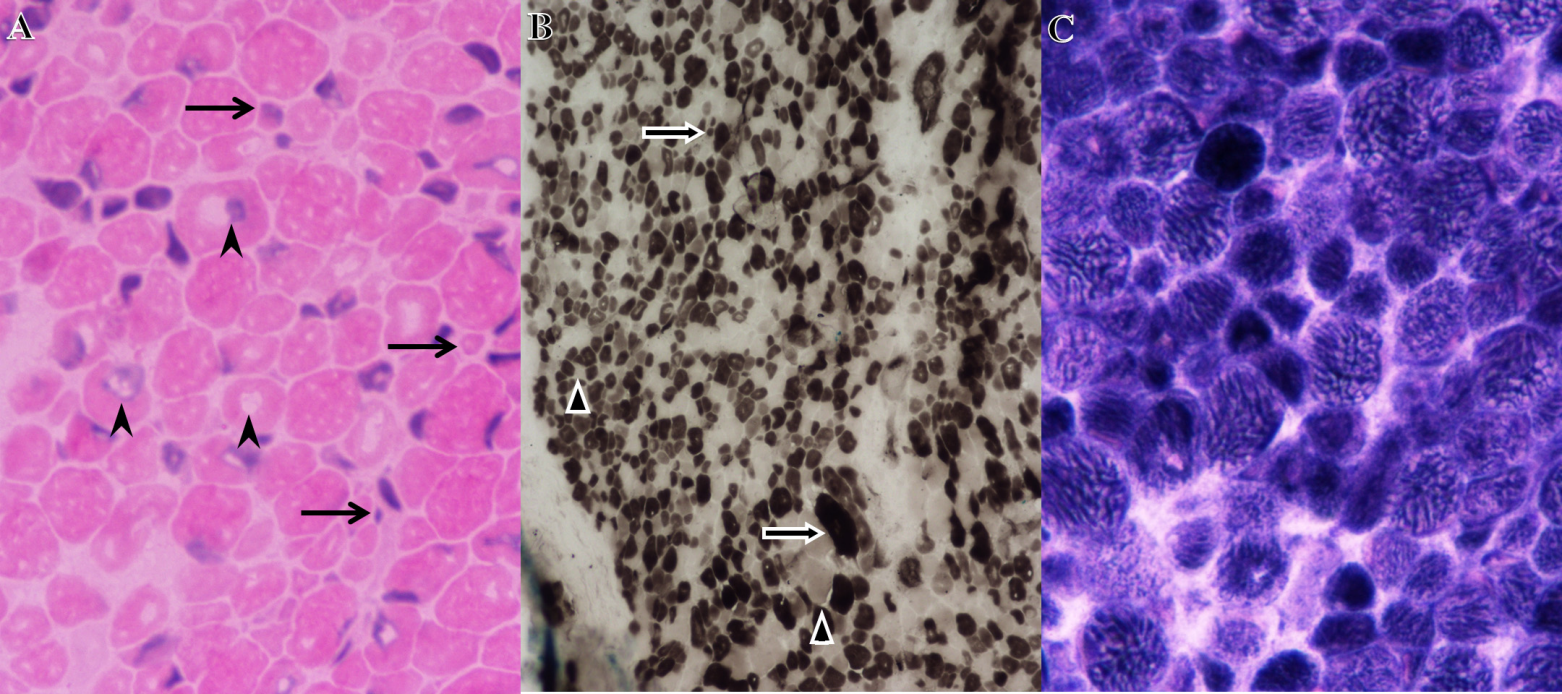
Histopathology images of frozen muscle biopsy cross sections from the affected male child in F045 carrying compound heterozygous p.Gln1649Glnfs*72 and p.Gln1265His *CACNA1S* pathogenic variants. (A) H&E stain demonstrating marked variability in myofiber size and fiber diameters ranging from 5 to 20 *μ*m. Arrows indicate tiny atrophic fibers. Central nuclei indicated by arrow points. Magnification = 600×. (B) ATPase reaction stain at pH 4.6 demonstrating that both fiber types are affected by hypertrophy and atrophy. Arrows indicate Type I (dark fibers) and arrowheads indicate Type II (light fibers). Magnification = 200×. (C) F-6-P stain demonstrating coarse myofibrillar architecture. Magnification = 600×.

The last case we describe was the product of a 38 week gestation of male–female twins conceived by IVF. The pair was born via C-section and the boy had APGAR scores of 8 and 9, but was described as being quite hypotonic, severely weak, not very vigorous, and never established the ability to significantly chew, suck, or swallow (Table1). The sister had no noted problems. The boy was hospitalized for 2 months and underwent extensive neurodiagnostic evaluation. During this time, a gastrostomy tube was inserted. His height, weight, and basic vital signs have remained normal throughout his course. At his evaluation at 3 month of age, he was somewhat dolichocephalic and plagiocephalic, for which he wore a helmet. He had a high-arched palate with mild micrognathia. He had clear pharyngeal phase dysphagia, oropharyngeal weakness, significant facial diplegia, but no apparent ptosis. He had no fixed contractures, but did have some flexion in his lower extremities (Table1). He was alert and responded to his parents’ voice and followed them visually, but he moved his head more than his eyes clearly demonstrating ophthalmoplegia. He had spontaneously movement of his hands and feet. Deep tendon reflexes were not obtainable. Muscle biopsy and extensive histology demonstrated moderate myofiber atrophy and hypertrophy of both fiber types with myofiber size variation ranging from 5 to 20 microns (Fig.6A). Fibers were polygonal with no myofiber degeneration or splitting. Occasional internal nuclei were present. No fiber type grouping and no inflammation or inclusions were seen (Fig.6B). Phosphofructokinase (F-6-P) staining revealed course myofibrillar architecture (Fig.6C). Other histological stains did not reveal any further abnormalities (Table1). High-resolution magnetic resonance imaging (MRI) did not reveal any structural abnormalities in the brain, cerebellum, and brainstem.

The boy, his parents, and his unaffected fraternal female twin were enrolled in our study. Analysis of variants identified by WES revealed novel pathogenic variants in *CACNAS1* in the family. The affected son received a pathogenic single bp deletion at g.chr1:201012509CT>C, c.4947delA, p.Gln1649Glnfs*72 (NM_000069.2) from his father and a missense g.chr1:201022587C>A c.3795G>T, p.Gln1265His (NM_000069.2) from his mother (Fig.3E). These variants were clinically confirmed by Sanger sequencing (Fig.4G and H). The missense p.Gln1265His variant is predicted to be damaging by FATHMM, Polyphen-2, mutationassessor (Reva et al. 2007, 2011), and MutationTaster. No prediction algorithms used assigned this variant as benign. These *CACNA1S* variants have not been reported in dbSNP, or ESP, but are each present in a single allele in ExAC (Table2). The twin sister of the affected boy was discordant for both *CACNAS1* variants (Fig.3E). The clinical features of the disease in Case 6 are consistent with a severe congenital myopathy with ophthalmoplegia. *CACNA1S* pathogenic variants have been identified as the cause of hypokalemic periodic paralysis type 1 (HOKPP1) (Burge and Hanna 2012; Hanchard et al. 2013), but persistent weakness of the child is somewhat inconsistent with HOKPP1. While it has been suspected that *CACNAS1* variants could cause myopathies due to the physical and mechanical coupling between its gene product, the voltage-dependent L-type calcium channel subunit alpha-1S (Cav1.1), and the Ryanodine receptor Ca^2+^ release channel 1 (RYR-1)(Wu et al. 2006; Rebbeck et al. 2014), no reports prior to this have identified pathogenic variants in *CACNAS1* as the cause of congenital myopathy.

## Discussion

Congenital and childhood myopathies and muscular dystrophies are a common class of neuromuscular disorders with overlapping phenotypes and heterogeneous genetic etiology. Here, we present the phenotype of 6 myopathy cases that underwent extensive, expensive, and invasive testing including muscle biopsies, EMG, MRI, EKG, and single gene and gene panel sequencing, often over many years, without successful genetic diagnosis prior to enrollment in our research study (Table1). We identified novel pathogenic variants in five genes by WES (Table2).

### Collagen 6 myopathies (cases 1-3)

We identified a recessive novel pathogenic variant in *COL6A3* and likely pathogenic variants in *COL6A6*. The family of Col6 proteins are extracellular matrix (ECM) proteins that help maintain tissue integrity of many tissues including muscle, tendon, skin, cartilage, and intervertebral disks (Chu et al. 1988; Knupp and Squire 2001; Bushby et al. 2014). The main forms of Col6 expressed in the ECM are the Collagen 6 alpha 1(Col6*α*1), 2(Col6*α*2), and 3(Col6*α*3) chains coded for by *COL6A1*, *COL6A2*, and *COL6A3*, respectively (Engvall et al. 1986; Bonaldo et al. 1998). Pathogenic variants in *COL6A1*, *COL6A2*, and *COL6A3* cause two main forms of myopathy; Ullrich congenital muscular dystrophy (UCMD) and Bethlem myopathy (BM) (Bonnemann 2011). Recently, the *COL6A6* gene was discovered. It is most similar to *COL6A3* as it codes for multiple von Willebrand factor type A (VWFA) domains (Gara et al. 2008; Tagliavini et al. 2014). *COL6A6* is expressed in wide range of fetal and adult tissue including brain, heart, and muscle (Fitzgerald et al. 2008; Gara et al. 2011). Col6*α*3 and Collagen 6 alpha 6(Col6*α*6) proteins are found in the endomysium and perimysium of skeletal muscle but only Col6*α*3 is found in the basement membrane (Sabatelli et al. 2011, 2012). Recent evidence demonstrates that Col6*α*6 is dramatically decreased in skeletal muscle and muscle cell cultures from patients with UCMD and BM independent of clinical phenotype suggesting coregulation of these genes (Tagliavini et al. 2014). Col6*α*6 was increased in noncollagen myopathies suggesting a significant role in other myopathies as well (Tagliavini et al. 2014).

Characteristic UCMD presents with severe congenital muscle weakness with axial and proximal joint contractures and distal joint hypermobility. BM usually presents with slowly progressive axial and proximal muscle weakness with finger flexion contractures. Skin rashes often accompany BM and UCMD (Bonnemann 2011; Bushby et al. 2014). Many pathogenic variants in Collagen 6 genes (*COL6*) impair protein expression by disrupting splicing, glycine substitutions required for triple-helix formation, or secretion. However, some pathogenic variants in BM patients have no detectable effect on Col6 assembly and secretion but compromise protein function in the ECM of muscle. Pathogenic variants in *COL6* genes have been reported as dominant and recessive (Lampe et al. 2008; Butterfield et al. 2013). The three cases describe here all display recessive phenotype since no carriers have yet displayed a phenotype. Interestingly, all three patients have the pathogenic *COL6A3* p.Lys2483Glu variant first reported by Brinas et al. (2010). Case 1 is homozygous for this variant. Case 2 is compound heterozygous for the p.Lys2483Glu variant and a *COL6A3* p.Gly1827Valfs*1 frameshift variant that would likely result in truncation and loss of expression. The phenotype of case 1 and case 2 are remarkably similar and indicate a myopathy more akin to the more mild BM form of disease. Their mild phenotype is very similar to that reported by Brinas et al. (2010) with the exception that our case demonstrated contractures. The F041 case had some additional features apart from the other two and is more suggestive of a more severe form of UCMD. The affected child carries novel compound heterozygous variants in *COL6A6* (p.Met2Lys and p.Cys1732Arg) in addition to being a heterozygous carrier of the *COL6A3* p.Lys2483Glu pathogenic variant. The *COL6A6* variants are likely pathogenic and we believe are the primary drivers of the phenotype, but the *COL6A3* variant likely contributes to the phenotype due to the overlap in expression and function. While it is clear there is a genetic component to her condition, we cannot rule out that complications at birth did not also contribute to her more severe phenotype.

It is clear that the *COL6A3* p.Gly1827Valfs*1 variant in F049 is pathogenic and is predicted to result in NSMD or protein truncation. The p.Lys2483Glu pathogenic variant in *COL6A3* causes a change in amino acid structure and opposite charge. It is located in the nonhelical region of the protein, in the VWFA 11 domain (Pan et al. 1998). Missense mutations in VWFA domains have been reported to cause BM (Pan et al. 1998). Changes in *COL6A3* expression or function of the p.Lys2483Glu variant at the endomysium and the perimysium is likely related to ECM proliferation identified in histology of F038 and F049 (Sabatelli et al. 2011; Bushby et al. 2014). The histology report for F049 indicates Col6 is present suggesting that the p.Lys2483Glu variant may not result in loss of expression or secretion, however, this must be considered cautiously as the pathology report does not indicate which Col6 proteins are detected. In the Brinas et al. (2010) case, Col6 was detected but altered in fibroblasts and not detected in muscle. In *COL6A6*, the p.Met2Lys missense pathogenic variant is located in the signal peptide of the protein and may disrupt its secretion. This variant may also disrupt translation of the *COL6A6* mRNA as mutations in stem loop structures near the start codon of other collagen genes abolish expression (Manojlovic and Stefanovic 2012). The p.Cys1732Arg pathogenic variant in *COL6A6* results in an addition of a charge to the amino acid and a change from hydrophobic to hydrophilic states. This variant is also located at the end of the triple-helical domain and just before the C-terminal VWFA domains (Pan et al. 1998). In general, Cys residues are highly conserved and are critical for formation of collagen oligomer and fibril disulfide bonds (Butterfield et al. 2013).

Of particular interest is the specific period of lipoatrophy and weight loss, and the inability to gain weight in cases 1 and 2, a characteristic of patients not typically described in cases of UCMD or BM (Brinas et al. 2010; Bonnemann 2011; Bushby et al. 2014), but which may be a diagnostic feature of disease caused by these variants. Weight loss and fatigue may be due to nocturnal respiratory insufficiency or apnea common to BM cases with *COL6A3* pathogenic variants (Bonnemann 2011). While suggested in case 2, neither case 1 nor 2 demonstrated waking respiratory insufficiency upon testing. Case 2 suffered from intermittent sleeping problems, but a sleep study did not reveal any apnea. Alternatively, several papers demonstrate metabolic changes in UCMD and BM including mitochondrial deficits in mouse models, cell models from UCHM and BM patients, and in patient muscle biopsies (Tagliavini et al. 2013; De Palma et al. 2014; Sorato et al. 2014). While muscle biopsy histology and EM did not reveal defects in morphology or number of mitochondria, respiratory chain deficits may explain the weight loss and fatigue seen in these patients.

Together, these three cases provide substantial evidence of the identification of pathogenic variants in *COL6A3* and likely pathogenic variants in *COL6A6*. While the *COL6A3* p.Lys2483Glu pathogenic variant is rare, it has a relatively high heterozygous frequency (Table2) in the population. Therefore, it is critical to report this pathogenic variant and it is very likely that many more genetically undiagnosed cases of BM will be found to have this variant.

### Emery–Dreifuss muscular dystrophy (case 4)

In addition to novel Col6 myopathies, we describe a novel c.187+1G>T pathogenic *EMD* splice variant in a single case of EDMD. EDMD usually manifests in childhood with slowly progressive weakness and limb muscle wasting. Contractures of the elbows, Achilles tendons, and postcervical muscles, are early and characteristic features. Cardiac conduction defects including arrhythmias and risk of sudden death are consistent features as well (Emery 1989; Bonne et al. 1993). The phenotype of the affected child in F043 is consistent with EDMD. The pathogenic variant we identified destroys the required canonical GT nucleotides of the 3′ splice site of exon 2 of the *EMD* gene. While pathogenic variants have been identified throughout the *EMD* gene, exon 2 has been reported as a mutational hotspot (Brown et al. 2011). This c.187+1G>T splice variant is also at the exact position of a previously reported c.187+1G>A pathogenic variant in EDMD (Deymeer et al. 1993; Yates et al. 1993, 1999). The G>A and G>T splice variants undoubtedly have very similar if not identical effects on *EMD* gene splicing and expression. The phenotype of F043 is consistent with phenotypes reported for families with the G>A splice variant (Deymeer et al. 1993; Yates et al. 1993, 1999). The identification of a second variant at the same genomic locus in individuals affected with EDMD confirms both variants as pathogenic.

F043 also demonstrates the importance of obtaining a genetic diagnosis for myopathy patients. In EDMD, cardiac dysfunction is a primary cause of premature mortality if left unmonitored and untreated. As described here, children with similar phenotypes (BM and EDMD) have very different requirements when it comes to cardiac assessment and treatment.

### *RYR1* and *CACNA1S* calcium channel myopathies (cases 5-6)

Finally, we describe two calcium channel myopathy cases. The first is a dominant case caused by a novel p.Ile4926ins7 pathogenic variant in the well-known CCD gene *RYR1*. Second, we describe a novel severe form of congenital myopathy with ophthalmoplegia caused by compound heterozygous pathogenic variants in *CACNA1S* (p.Gln1649Glnfs*72 and p.Gln1265His). Several calcium channelopathies are associated with pathogenic variants in *RYR1* and *CACNA1S*. Malignant Hyperthermia Susceptibility (MHS) is caused by pathogenic variants in *RYR1* or *CACNA1S* (Monnier et al. 1997; Kim et al. 2013). Pathogenic variants in *CACNA1S* have been identified as a cause of HOKPP1, but *RYR1* have not (Burge and Hanna 2012; Hanchard et al. 2013). Pathogenic variants in *RYR1* are the most common causes of CCD, while *CACNA1S* have not been identified as a cause of CCD (Wu et al. 2006). CCD is most often characterized clinically by a stable or slowly progressive course of congenital hypotonia but can be more severe (Bharucha-Goebel et al. 2013). Most *RYR1* pathogenic variants usually result in a dominant phenotype consistent with the dominant heterozygous p.Ile4926ins7 pathogenic variant identified by WES in F047. Of special interest in this case is that the pathogenic variant in the affected father is *de novo*, which he then passed on to his affected son.

*RYR1* and *CACNA1S* genes code for integral components of excitation-contraction coupling (EC) in skeletal muscle. EC depends on a physical interaction between the skeletal forms of the dihydropyridine receptor L-type Ca^2+^ channel (DHPR) and RYR-1 (Paolini et al. 2004; Polster et al. 2012). The *CACNA1S* gene codes for Cav1.1, the main subunit of the DHPR channel (Rebbeck et al. 2014). When a voltage neurostimulus is received, the DHPR channel changes conformation and physically causes RYR-1 to open and release sarcoplasmic reticulum calcium (Rebbeck et al. 2014).

The F047 p.Ile4926ins7 in *RYR1* is located in the final hydrophobic transmembrane domain of RYR-1 which has been designated as a pathogenic variant hotspot (Maclennan and Zvaritch 2011). This insertion is in-frame, likely expressed, and is predicted to result in addition of seven amino acids in the final transmembrane helix nearest the Ca^2+^ pore formed by RYR-1. These extra amino acids undoubtedly alter RYR-1 function and folding. Since this variant shows dominant inheritance, it likely has a dominant negative effect by poisoning RYR-1 tetramer formation and function (Maclennan and Zvaritch 2011).

In F045, the severe congenital myopathy with ophthalmoplegia strongly suggested pathogenic variants in *RYR1*, but no pathogenic variants in *RYR1* were identified. Instead, we identified novel pathogenic frameshift (p.Gln1649Glnfs*72) and missense (p.Gln1265His) variants in *CACNA1S*. The frameshift variant likely abolishes translation of the mRNA. If the resulting mRNA is translated to protein, it is predicted to result in premature truncation later in the protein. Cav1.1 has four transmembrane domains each with six transmembrane helices that form the Ca^2+^ channel. The Cav1.1 p.Gln1265His pathogenic variant is located in its fourth repeat domain in the short cytoplasmic loop linking the transmembrane helix S4 and the positively charged transmembrane helix S5. This cytoplasmic loop may be involved in interactions with RYR-1 or other components of the DHPR (Rebbeck et al. 2014). The change in amino acid structure may also interfere with DHPR calcium flux. Due to the phenotypic overlap with *RYR1* pathogenic variants in F045, we hypothesize that the p.Gln1265His variant disrupts DHPR and RYR-1 coupling. While pathogenic variants in *CACNA1S* have been demonstrated as the cause of MHS and HOKPP1, to our knowledge, this is the first reported case of severe congenital myopathy with ophthalmoplegia resulting from pathogenic variants in *CACNA1S*.

Reporting these cases not only raises awareness to extensive clinical overlap between similar cases with different genetic etiology but most importantly highlights the utility of WES and WGS in providing genetic diagnosis in clinically enigmatic cases.

## Supporting Information

**Figure S1.** Exome target coverage statistics. The percent of bases of the targeted exome that had at least 20 high quality reads covering each base.
