# Supplementary figures and images for: Novel pathogenic variants and genes for myopathies identified by whole exome sequencing
Source: Mol Genet Genomic Med. 2015 Apr 8;3(4):283–301. doi: 10.1002/mgg3.142 (PMC4521965; doi:10.1002/mgg3.142)

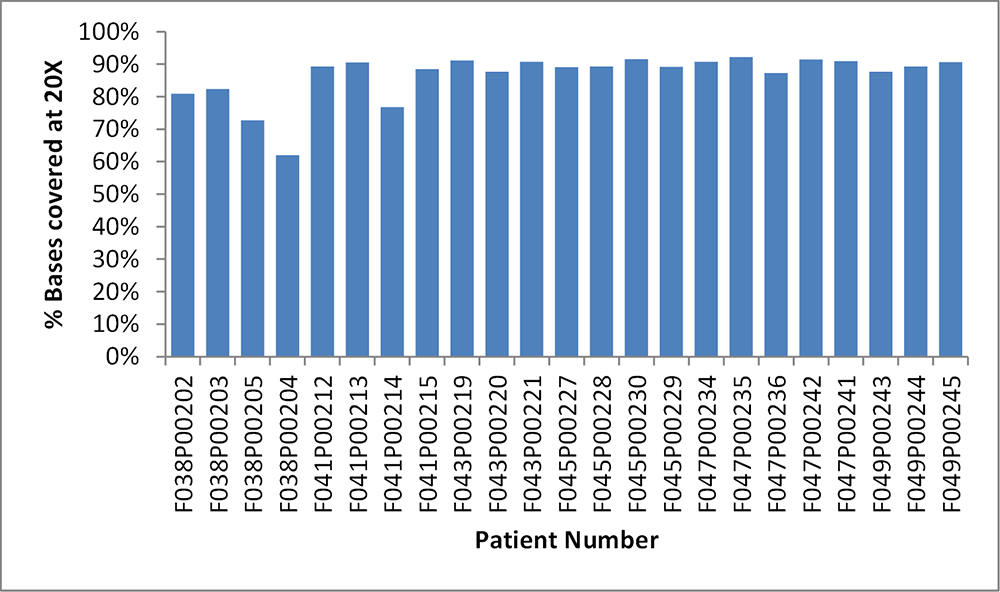

Supplement: Supplementary file 1 [file mgg30003-0283-sd1.tif]
